# Prevalence and microbiological profile of septic complications following ECMO decannulation: a prospective single-center study

**DOI:** 10.1038/s41598-025-34085-1

**Published:** 2025-12-29

**Authors:** Simone Kattner, Ksenia Pawlytta, Andrea Engler, Frank Herbstreit, Ivana Kraiselburd, Folker Meyer, Yevhen Vainshtein, Mirko Sonntag, Kai Sohn, Thorsten Brenner, Marcel Hochreiter

**Affiliations:** 1 Department of Anesthesiology and Intensive Care Medicine, University Hospital Essen, University Duisburg-Essen, Essen, Germany; 2 Institute for Artificial Intelligence in Medicine, University Hospital Essen, University Duisburg-Essen, Essen, Germany; 3 Innovation Field In-Vitro Diagnostics, Fraunhofer Institute for Interfacial Engineering and Biotechnology IGB, Stuttgart, Germany; 4 Department of Anesthesiology and Intensive Care Medicine, Westcoast Hospital, Heide, Germany

**Keywords:** ECMO, Sepsis, Decannulation, Bacteremia, Intensive care, cfDNA, Diseases, Medical research, Microbiology

## Abstract

Extracorporeal membrane oxygenation (ECMO) is widely used to manage acute respiratory distress syndrome (ARDS); however, biofilm formation on cannulas may contribute to infections. This study investigated the prevalence, timing, and microbial profiles of infectious complications following ECMO decannulation. This prospective, single-center cohort study was conducted in the mixed medical-surgical intensive care unit (ICU) at the University Hospital Essen (01/2022–01/2023). Adults who received ECMO for > 48 h were included. Microbiological sampling (blood cultures and wound swabs) was performed before and after decannulation. Cannulas were assessed for biofilms, and plasma samples were analyzed using next-generation sequencing (NGS) of microbial cell-free DNA (cfDNA). Sepsis was defined according to the most recent Sepsis-3 criteria. The study included 18 patients (56% men); 17 received VV-ECMO and one received VA-ECMO. Post-decannulation sepsis occurred in 10 of 18 patients (56%; 95% CI: 31–79%), and overall infectious complications were observed in 72% of patients. A strong negative correlation was evident between pre-ECMO ventilation duration and biofilm-forming bacteria in the blood during ECMO (*r* = − 0.7, *p* = 0.002). Blood cultures obtained within 10 min of decannulation were positive in 6 of 18 (33%) patients. NGS identified pathogens in 9 of 12 patients (75%), with 5 (42%) revealing additional organisms not detected by conventional methods. Viral pathogens were detected in 3 of 12 (25%) patients using NGS. Patients with sepsis demonstrated higher antibiotic consumption (*p* = 0.012) and more frequently met SOFA-based sepsis criteria (*p* = 0.007), whereas other parameters were comparable. Sepsis and infection frequently occur after ECMO decannulation and may be associated with biofilm-related pathogens. NGS has improved pathogen detection beyond standard diagnostics, although the findings require clinical correlation. These prospective findings support the need for further investigation of advanced integrated microbiological surveillance post-ECMO in larger multicenter studies.

**Trial registration**: DRKS, DRKS00024842. Registered 12 Apr 2021, https://drks.de/search/de/trial/DRKS00024842.

**Supplementary Information:**

The online version contains supplementary material available at 10.1038/s41598-025-34085-1.

## Introduction

Extracorporeal membrane oxygenation (ECMO) has emerged as a vital life-support technology for patients with severe respiratory or cardiac failure refractory to conventional treatment. Since its inception in the 1970s, ECMO has evolved significantly, with improvements in technology and management strategies leading to increased utilization in intensive care units (ICUs) worldwide^1^. The coronavirus disease 2019 (COVID-19) pandemic further highlighted ECMO’s potential, as it became a last-resort therapy for patients with severe acute respiratory distress syndrome (ARDS) caused by severe acute respiratory syndrome coronavirus type 2 (SARS-CoV-2) infection^2,3^.

Despite its life-saving potential, ECMO is associated with significant complications, with infections being one of the most concerning^4^. The presence of large-bore cannulas, prolonged ICU stays, and the immunomodulatory effects of extracorporeal circulation all contribute to the increased risk of nosocomial infections in patients undergoing ECMO^5,6^. These infections range from localized cannula-site infections to sepsis or septic shock, significantly affecting patient outcomes and healthcare resource utilization following ECMO treatment^7,8^.

Sepsis, defined as life-threatening organ dysfunction caused by a dysregulated host response to infection^9^, poses a challenge for patients undergoing ECMO^10^. Clinical scoring metrics, such as the Sequential Organ Failure Assessment (SOFA) score, are important for the early identification of sepsis. However, the diagnosis of infection in ECMO-treated patients is complicated by several factors. ECMO can induce a systemic inflammatory response that mimics sepsis^11^. Moreover, traditional markers of infection and organ dysfunction, such as procalcitonin (PCT), C-reactive protein (CRP), or leukocyte count, may be altered by ECMO^12^. The hemodynamic effects of ECMO can mask the signs of septic shock^13^. Moreover, the management of sepsis in patients undergoing ECMO is complex, requiring a careful balance of antimicrobial therapy, fluid management, catecholamines, and adjustment of ECMO settings^14,15^.

Despite the growing use of ECMO and the recognized importance of ECMO-related infectious complications, several knowledge gaps remain. The true incidence and timing of sepsis in patients on ECMO, particularly post-decannulation, remain unclear^16^. In 2024, approximately 63,876 ECMO connections for pulmonary failure and an additional 66,402 for cardiac failure were recorded worldwide in adults by the Extracorporeal Life Support Organization (ELSO)^17^. For ECMO therapy, two large-lumen cannulas are placed either in two large veins (VV-ECMO) or in one vein and one artery (VA-ECMO).

Apart from ECMO cannulas, several other catheters are inserted into the bloodstream of these patients for routine intensive care therapy. Microorganisms can deposit and form biofilms on the surfaces of these cannulas or catheters^18^. Biofilms can also form on other medical implants^19^. The entry points of this microorganism can be the insertion site, catheter hub, infusion fluids, non-intact dressing, or hematogenous spread. The most common biofilm-forming bacteria in humans are *Enterococcus* spp., *Escherichia (E.) coli*, coagulase-negative *Staphylococci*, *Klebsiella (K.) pneumoniae*, *Pseudomonas (P.) aeruginosa*, *Staphylococcus (S.) aureus* und *Proteus (Pr.) mirabilis*^18^, as well as the fungus Candida (C.) albicans. Patients have been reported to have positive blood cultures before or after the initiation of ECMO therapy^20^. Several studies have reported the presence of biofilms on ECMO cannulas^21,22^. These biofilms are assumed to be formed by pathogens of bloodstream infections before or during ECMO therapy^23^. In addition, these biofilms may exfoliate during ECMO decannulation and enter the bloodstream of patients, potentially contributing to sepsis^24^.

The microbiological profile of ECMO-associated infections, including the role of biofilm-forming organisms, has not been well characterized^25^. Furthermore, the relationship between pre-ECMO factors, ECMO duration, and post-ECMO sepsis remains unclear^26^. Optimal strategies for the diagnosis and management of sepsis in the immediate post-ECMO period have not been established^27^. As ECMO continues to play a crucial role in the management of critically ill patients, particularly in the context of global health crises, a deeper understanding of its associated infectious complications is vital for improving overall patient care and outcomes in intensive care settings^28^. Such advancements could significantly impact patient outcomes, reduce healthcare costs, and optimize resource utilization in high-risk populations^29^.

Conventional blood cultures remain the diagnostic standard for bloodstream infections but have well-recognized limitations: prolonged time-to-result (24–72 h), reduced sensitivity in patients receiving antibiotics, and inability to detect fastidious or nonculturable organisms^30^. Next-generation sequencing (NGS) of microbial cell-free DNA (mcfDNA) offers a culture-independent approach capable of detecting bacterial, fungal, viral, and parasitic DNA directly from plasma within 24 h^31^. This method has demonstrated improved pathogen detection in patients with sepsis compared to conventional cultures, particularly in those receiving antimicrobial therapy^32^. Given that most ECMO patients receive antibiotics prior to decannulation, mcfDNA sequencing may provide additional diagnostic information in this population.

The present prospective study aimed to determine the prevalence and timing of infectious complications following ECMO decannulation, characterize the microbiological profile based on blood culture and mcfDNA sequencing of post-ECMO infections, and investigate the relationship between pre-ECMO factors, ECMO duration, and post-ECMO sepsis.

## Methods

### Study design and participants

**Fig. 1:**
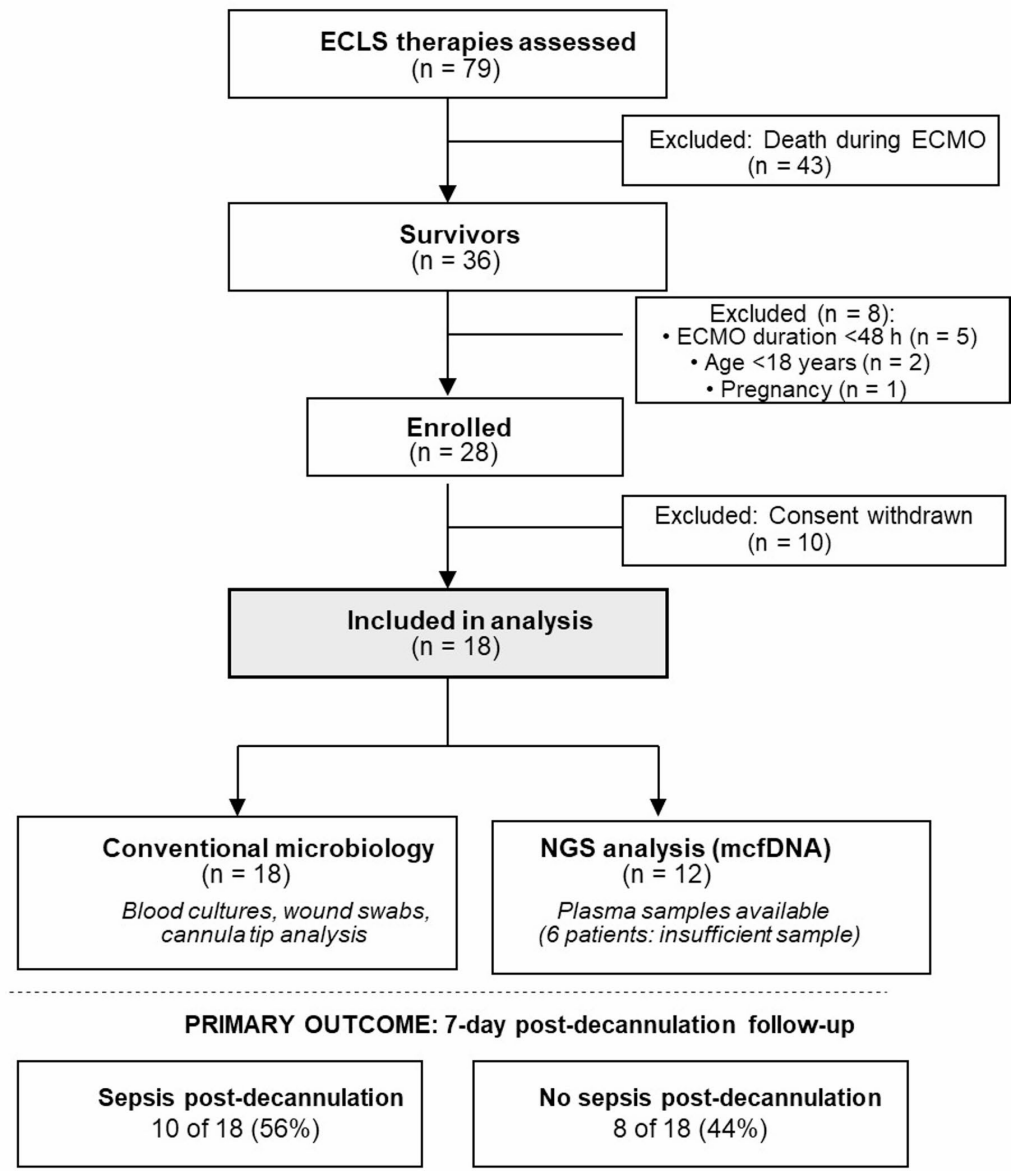
Patient enrollment and study flow.

This prospective, single-center cohort study was conducted in the mixed medical-surgical ICU of University Hospital Essen, Germany, a supraregional referral center for respiratory and cardiac ECMO therapy, between Jan 2022 and Jan 2023. Adults (aged ≥ 18 years) receiving VV- or VA-ECMO for > 48 h were eligible for inclusion in the study. The exclusion criterion was pregnancy (Table S1). This study was approved by the Ethics Committee of the University Hospital Essen (approval number: 20-9637-BO). The patient flow is summarized in Fig. 1.

Informed consent was obtained using a tiered method. Capable patients provided direct consent, whereas legal representatives consented to the consent of incapacitated patients. For patients initially lacking capacity, an independent external intensive care physician evaluated study eligibility, and these patients were subsequently informed and provided consent after regaining decision-making ability. Only patients who provided full informed consent were included in the final analysis.

### Procedures

VV-ECMO was initiated for ARDS in accordance with the established guidelines^33^. VA-ECMO was employed for refractory cardiogenic shock despite optimal catecholamine therapy, defined as norepinephrine ≥ 0.5 µg/kg/min with mean arterial pressure < 65 mmHg despite adequate fluid resuscitation. Percutaneous cannulation for both VA- and VV-ECMO was performed under sterile conditions using HLS cannulas (Maquet, Hirrlingen Germany). Skin antisepsis protocols evolved during the study period: an alcohol-based antiseptic (Kodan ^®^ Tinktur forte colored) was used until Nov 2022, after which Octeniderm^®^ (octenidin 0.1%) was employed. Sterile dressings were changed daily during routine care.

Anticoagulation during ECMO was managed according to the institutional protocols. For VV-ECMO, unfractionated heparin was titrated to a target activated partial thromboplastin time (aPTT) of 45–60 s or anti-Xa level of 0.2–0.3 IU/mL. For VA-ECMO, higher anticoagulation targets were employed (aPTT 60–80 s, anti-Xa 0.3–0.5 IU/mL)^34^. Anticoagulation was held for 4–6 h prior to decannulation, when clinically feasible.

Sedation was managed to achieve a target Richmond Agitation-Sedation Scale (RASS) score of − 2 to 0 when clinically feasible. Deeper sedation (RASS − 3 to − 5) was employed during prone positioning for patients with severe respiratory failure requiring lung-protective ventilation or for the management of agitation and delirium. The observed RASS of − 3 on the decannulation day (Table 2) reflects the procedural sedation requirements and clinical status of patients at this transition point.

Microbial sampling was performed at various time points in this study. Before decannulation, one pair of blood cultures was obtained via peripheral venous puncture or new catheter insertion. Swabs were obtained from inflamed cannula sites, when present. Within ten minutes post-decannulation, additional blood cultures were drawn, and the first 5 cm of the ECMO cannula tips were collected and rinsed with sterile 0.9% NaCl. The Institute of Microbiology, University Hospital Essen, performed all microbiological analyses, including aerobic and anaerobic bacterial and fungal cultures and antimicrobial susceptibility testing for positive results. In 12 patients, plasma was collected parallel to the study blood cultures before and after ECMO decannulation. These were analyzed for mcfDNA using next-generation sequencing (NGS) by the Fraunhofer Institute for Interfacial Engineering and Biotechnology IGB, Stuttgart, Germany.

Biofilm-forming bacteria were defined as organisms commonly associated with biofilm formation on medical devices, including *Enterococcus* spp., *E. coli*, coagulase-negative staphylococci, *K. pneumoniae*, *P. aeruginosa*, *S. aureus*, *Proteus mirabilis*, and *C. albicans*^18^. Fungal detection refers to positive culture for fungal organisms (*Candida* spp. and *Aspergillus* spp.) from normally sterile sites (blood, bronchoalveolar lavage, and pleural fluid) or from urine/tracheal aspirates with clinical correlation.

This prospective study collected plasma samples at defined time points. Conventional microbiological results were available in real time and guided clinical management. NGS analysis of stored plasma was performed retrospectively; the results were not available during patient care and therefore did not influence antimicrobial decisions.

Patients were monitored for septic complications for seven days after ECMO decannulation. All patient data, laboratory values, and microbiological test results were extracted from patient files (Table S2). Standard therapy was administered according to the protocol of the attending physician.

### Cell-free DNA (cfDNA) isolation

Patient blood samples were drawn, and plasma was harvested as described by Brenner et al. (2018)^35^. Briefly, whole blood was centrifuged for 10 min at 1600 × g and 4 °C. The plasma supernatant was obtained and centrifuged for an additional 10 min at 16,000 × g and 4 °C. Subsequently, 1.1 mL of the plasma supernatant was transferred into a fresh 1.5 mL DNA LoBind tube and stored at − 80 °C. Before cell-free DNA (cfDNA) isolation, plasma samples were centrifuged for 10 min at 10,000 × g at 4 °C to remove residual cells and debris. For cfDNA isolation, the QIAamp MinElute ccfDNA Kit (Qiagen, Germany) was used with 1 mL of plasma input according to the manufacturer’s protocol, with the exception of cfDNA elution in 60 µL nuclease-free water (Ambion, USA). For quantification of eluted cfDNA, the concentration was measured using a Qubit dsDNA HS assay kit (Thermo Fisher Scientific, USA), and the cfDNA quality was assessed using a Fragment Analyzer high-sensitivity DNA kit (Agilent, USA).

### Library preparation and high-throughput sequencing

Sequencing libraries for cfDNA were prepared using the NEXTFLEX cell-free DNA-seq kit V2 (PerkinElmer, USA) according to the manufacturer’s instructions. Library generation was performed using a Biomek FXP workstation (Beckman Coulter). As input, 10 ng of cfDNA was used, with the exception of the negative control, where 32 µL of eluted cfDNA was used (2.5 ng input). The resulting cfDNA sequencing libraries were eluted in 20 µL of nuclease-free water. Library quality was assessed using the fragment analyzer high-sensitivity DNA kit (Agilent), and the concentration was measured using the Qubit dsDNA HS assay kit (Thermo Fisher Scientific, USA), as described in the previous section. Sequencing was performed on a NextSeq 2000 (Illumina, USA) with 100 bp single-end reagent kits, aiming for at least 30 million raw reads per sample.

### Bioinformatic processing of sequencing data

Sequencing data were demultiplexed using bcl2fastq (v2.20.0.422). Quality control (QC) of both raw and processed reads was performed with FastQC (v0.12.1). Adapter removal and quality trimming were conducted using BBDuk from the BBMap package (v39.01) with the following parameters: trimpolyg = 10, hdist = 1, mink = 12, threads = 20, maxns = 10, minlen = 50, trimq = 20, qtrim = t, ktrim = r, k = 28.

Human-derived reads were filtered out after trimming by mapping against the human reference genome GRCh38 using NextGenMap (v0.5.5). Unmapped reads were extracted using samtools (v1.6), and subsequently converted from BAM to FASTQ format using bamtools (v2.5.1). Low-complexity sequences were removed using PRINSEQ-lite (v0.20.4).

The taxonomic classification of the remaining non-human reads was performed using Kraken2 with a custom-built reference database. This database includes complete genomic sequences from bacteria, viruses, fungi, parasites, and plants, as well as entries from the NCBI RefSeq database. Only species-level classifications with confidence scores ≥ 0.6 were retained.

Post-classification analyses were performed using the in-house Python scripts. For clinical interpretation, the classified reads were further processed to compute a Sepsis Indicating Quantifier (SIQ) score with an R script, using a reference cohort of non-septic postoperative patients from the NextGeneSiS study. The SIQ scoring computational pipeline is described in detail by Grumaz et al. (2016)^36^. A comprehensive description of the control cohort used for calibration is available in the recent publication by Brenner et al.(2025)^37^. It should be noted that the detection of mcfDNA indicates circulating microbial DNA but does not confirm viable organisms or active bloodstream infections. Potential sources of false-positive results include reagent contamination, sample handling, and circulating DNA from non-viable organisms or translocated gut flora. To distinguish NGS findings from culture-confirmed bacteremia, the term “DNAemia” is used when referring to mcfDNA detection.

### Outcomes

The primary outcome was the prevalence of septic complications following ECMO decannulation. Sepsis was defined as an increase in SOFA score by ≥ 2 points, and septic shock was defined as sepsis with vasopressor requirement to maintain a mean arterial pressure ≥ 65 mmHg and serum lactate > 2 mmol/L (> 18 mg/dL) in the absence of hypovolemia, according to the Sepsis-3 criteria modified for ICU patients as described by Vasilevskis et al.^38,39^ (Tables S3 and S4 in the Supplement). Secondary outcomes included microbiological findings and decannulation-related bacteremia, defined as at least one positive blood culture after ECMO decannulation.

### Statistical analysis

Normal distribution was assessed using the Shapiro-Wilk test. Normally distributed continuous variables are presented as mean (standard error), and non-normally distributed variables are presented as median (interquartile range). Categorical variables are presented as numbers (percentages) with 95% confidence intervals (CI) for key proportions. Paired t-tests or Wilcoxon rank tests were used for continuous variables, and McNemar or Fisher’s exact tests were used for categorical variables. The Pearson correlation coefficient was used to assess the relationships between pre-ECMO hospital stay, ICU stay duration, ECMO therapy duration, and post-ECMO sepsis occurrence. Statistical significance was set at *p* < 0.05, and all tests were 2-tailed.

## Results

### Patient flow

Between Jan 2022 and Jan 2023, 79 ECLS therapies were performed at our institution (Fig. 1). Of these, 43 (54%) died during ECMO support. Among the 36 survivors, eight were excluded: five due to ECMO duration < 48 h, two due to age < 18 years, and one due to pregnancy. Twenty-eight patients met the inclusion criteria and were enrolled in the study. Subsequently, 10 patients withdrew their consent after regaining their decision-making capacity, leaving 18 patients for the final analysis. All 18 patients underwent conventional microbiological testing for diagnosis. Plasma samples for NGS analysis were available for 12 patients; 6 patients had insufficient sample volumes for mcfDNA sequencing.

### Characteristics of study population

#### Demographics, comorbidities and ECMO-indications

From Jan 2022 to Jan 2023, 79 ECLS therapies were performed, of which 36 survived, and 28 were included in the study. Of these, 10 patients did not provide definitive consent and were subsequently excluded. The study included 18 patients, of whom 10 (56%) were male (Table 1). VV-ECMO was performed in 17 patients, whereas VA-ECMO was performed in one patient. The comorbidities included COPD (*n* = 3), bronchial asthma (*n* = 5), interstitial lung disease (*n* = 1), and lung transplantation (*n* = 1). Six patients received immunosuppressive medications prior to ECMO initiation. The most common reason for ECMO therapy was ARDS due to viral pneumonia, with SARS-CoV-2 (*n* = 6) and influenza A (*n* = 2) being the predominant causes. Other indications included bacterial pneumonia (*n* = 5), status asthmaticus (*n* = 3), fulminant pulmonary embolism (*n* = 1), and myocardial infarction (*n* = 1).

**Table 1:** Demographics and characteristics of all enrolled subjects.

Variables	All (*n* = 18)
Characteristics	
Age, yr	46 ± 3
Gender, M/F	10/8
BMI, kg/m^2^	29 ± 1
Pre-existing conditions	
Diseases, n	3 ± 1
Lung diseases, yes/no	10/8
Immunosuppression medication, yes/no	6/12
Duration of	
Intubation pre-ECMO, days	1 [0–3]
ECMO therapy, days	12 [6–26]
ICU stay, days	24 [22–37]
Reasons for acute respiratory failure	
ARDS	13 (72%)
Primary viral pneumonia	8 (44%)
Primary bacterial pneumonia	5 (28%)
Status asthmaticus	3 (17%)
Other reason for ECMO	2 (11%)
Infections	
Sepsis pre-ECMO therapy	12 (67%)
Sepsis during ECMO therapy	17 (94%)
Sepsis pneumogenic	15 (83%)
Antibiotics pre-ECMO therapy	12 (67%)
Antibiotics during ECMO therapy	17 (94%)
Microbiological evidence	
Bacterial detection pre-ECMO therapy	1 (6%)
Bacterial detection during ECMO therapy	14 (78%)
Bacterial detection after ECMO therapy	13 (72%)
Fungal detection pre-ECMO therapy	1 (6%)
Fungal detection during ECMO therapy	1 (6%)
Fungal detection after ECMO therapy	3 (17%)

Data are presented as mean ± SEM or median [IQR]. ARDS, acute respiratory distress syndrome; BMI, body mass index; ECMO, extracorporeal membrane oxygenation; ICU, intensive care unit; IQR, interquartile range; SEM, standard error of the mean.

### Infectious complications (including sepsis) after ECMO decannulation

Sepsis was present in 12 of 18 patients (67%) prior to ECMO initiation, in 17 of 18(94%) during ECMO, and in 10 of 18 patients (56% [95% CI: 31–79%]) after ECMO (Table 2). Infectious complications were observed in 13 patients (72%) after ECMO termination. A strong negative correlation was observed between the duration of mechanical ventilation prior to ECMO initiation and the detection of biofilm-forming bacteria during ECMO (*r* = -0.7, *p* = 0.002). Moreover, the duration of ECMO tended to be longer in cases with microbiological evidence of pathogens before ECMO (*r* = 0.6, *p* = 0.008). It should be noted that Table 2 reports conventional culture-based bacterial detection, whereas NGS findings are presented separately. Three patients in the non-septic group demonstrated positive blood cultures after decannulation without meeting the sepsis criteria, suggesting that transient bacteremia following decannulation does not invariably progress to sepsis. The added value of NGS was most evident in patients with sepsis, where conventional cultures were negative (patients 7, 10, 15, and 18), providing potential explanations for clinical deterioration.

Among these patients, two (11%) were already identified as septic on the day after ECMO decannulation (day x + 1) (patients 10 and 18), and eight (44%) developed sepsis within the subsequent 7 days (Tables 3, 4 and 5). Accordingly, the leukocyte count increased significantly (*p* = 0.04, Table 3), and body temperature tended to be higher (*p* = 0.07, Table 3) on day x + 1. Antibiotic use tended to increase after decannulation (*p* = 0.06, Table 3). In general, the assessment of infection complications in patients was slightly higher on day x + 1, despite a reduction in PCT and SOFA scores (*p* = 0.06, Table 3). Three additional patients exhibited clinical signs of infection and bacteremia; however, they did not meet the full criteria for sepsis. These improvements were noted in specific SOFA components, particularly in the coagulation, respiratory, and neurological system scores (Table S4).

**Table 2:** Comparison of patient characteristics: sepsis vs. non-sepsis after ECMO decannulation.

Variables	Non-sepsis (*n* = 8)	Sepsis (*n* = 10)	*p*-value
Characteristics			
Age, yr	51 ± 5	41 ± 4	0.100*
Gender, M/F	6/2	4/6	0.185
BMI, kg/m^2^	31 ± 2	27 ± 2	0.169*
Pre-existing conditions			
Diseases, n	3 ± 1	2 ± 1	0.508*
Lung diseases, yes/no	5/3	5/5	0.659
Immunosuppression medication, yes/no	3/5	3/7	1.000
Duration of			
Intubation pre-ECMO, days	1 [1–3]	1 [0–3]	0.315†
ECMO therapy, days	17 [6–28]	10 [6–23]	0.436†
ICU stay, days	29 [17–32]	23 [22–39]	0.912†
Reasons for acute respiratory failure			
ARDS, yes/no	6/2	7/3	1.000
Primary viral pneumonia, yes/no	3/5	5/5	0.659
Primary bacterial pneumonia, yes/no	2/6	3/7	1.000
Laboratory parameters			
Leucocyte, ×10^3^/µL, day x	13 ± 2	12 ± 2	0.626*
CRP, mg/dL, day x	17 ± 4	17 ± 4	0.921*
PCT, ng/mL, day x	0.4 [0.2–3.6]	1.9 [0.3–8.8]	0.529†
Lactate, mmol/L, day x	1.2 ± 0.1	1.2 ± 0.2	0.836*
Clinical parameters			
SOFA score, day x	11 ± 2	12 ± 1	0.510*
Sedation (RASS), day x	−3 ± 0.5	−3 ± 0.5	0.589*
Norepinephrine, µg/kg/min, day x	0.00 [0–0.01]	0.01 [0–0.1]	0.218†
PaO_2_/FiO_2_ ratio, day x	81 ± 4	111 ± 7	0.021*
Infections			
Sepsis pre-ECMO therapy, yes/no	5/3	7/3	0.500
Antibiotics during ECMO therapy, yes/no	8/0	9/1	0.556
Positive SOFA score (≥ 2 pt increase), until day x + 7, yes/no	0/8	10/0	0.007
Antibiotics after ECMO therapy, yes/no	1/7	10/0	0.012
Microbiological evidence			
Bacterial detection pre-ECMO, yes/no	0/8	1/9	1.000
Bacterial detection during ECMO, yes/no	6/2	8/2	0.500
Bacterial detection post-ECMO, yes/no	3/5	3/7	1.000

Data are presented as mean ± SEM or median [IQR]. *p-value by t-test; †p-value by Mann-Whitney U test; all others by Fisher’s exact test. Bacterial detection refers to conventional, culture-based microbiological testing. The NGS results are presented in Tables 4 and 5. ARDS, acute respiratory distress syndrome; BMI, body mass index; CRP, C-reactive protein; ECMO, extracorporeal membrane oxygenation; ICU, intensive care unit; IQR, interquartile range; PCT, procalcitonin; RASS, Richmond Agitation and Sedation Scale; SEM, standard error of the mean; SOFA, Sequential Organ Failure Assessment.

### Septic and non-septic patients after ECMO decannulation

No differences were observed between the septic and non-septic groups following ECMO decannulation in terms of demographic characteristics, comorbidities, duration of therapy, or underlying causes of acute respiratory failure (Table 2). Laboratory values and clinical parameters were comparable between the two groups. However, the PaO₂/FiO₂ ratio showed a more pronounced improvement in the non-septic group. Notably, antibiotic use (*p* = 0.012) and the proportion of patients meeting SOFA-based sepsis criteria during follow-up (*p* = 0.007) differed significantly between groups. Among patients who developed sepsis, 77% had a detectable bloodstream pathogen after decannulation, and all received antibiotic treatment.

**Table 3:** Laboratory parameters and scores.

Variables	Day x	Day x + 1	*p*-value
Laboratory parameters			
Leucocyte, ×10^3^/µL	12 ± 1	14 ± 1	0.041*
CRP, mg/dL	17 ± 3	16 ± 2	0.150*
PCT, ng/mL	1 [0–5]	1 [0–4]	0.043†
Lactate, mmol/L	1.2 ± 0.1	1.1 ± 0.1	0.896*
Clinical parameters			
SOFA score	12 ± 1	9 ± 1	0.001*
Sedation (RASS)	−3 ± 0.3	−2 ± 0.4	0.049*
Norepinephrine, µg/kg/min	0.01 [0–0.13]	0.06 [0–0.24]	0.317†
PaO_2_/FiO_2_ ratio	98 ± 6	257 ± 23	0.001*
Body temperature, °C	37 ± 0.2	38 ± 0.2	0.068*
Clinical assessment			
Sepsis (Day x + 1), yes/no	2/16	7/11	0.063
Sepsis post-ECMO, yes/no	2/16	14/4	0.001
Antibiotic therapy, yes/no	8/10	13/5	0.063
Microbiological detection			
Bacteria in blood cultures, yes/no (*n* = 17)‡	2/15	6/11	0.125
Positive cannula, yes/no (*n* = 9)§	–	1/8	–

Data are presented as mean ± SEM or median [IQR]. *p-value by paired t-test; †p-value by Wilcoxon test; all others by McNemar’s test. ‡ Blood cultures obtained prior to (Day x) and within 10 min after (Day x + 1) ECMO decannulation. One patient (Patient 3) did not have blood cultures obtained; therefore *n* = 17 for blood culture analyses. § ECMO cannula tips were microbiologically analyzed in 9 of 18 patients. One cannula was positive (S. epidermidis, Patient 15); eight were negative. CRP, C-reactive protein; ECMO, extracorporeal membrane oxygenation; IQR, interquartile range; PCT, procalcitonin; RASS, Richmond Agitation and Sedation Scale; SEM, standard error of the mean; SOFA, Sequential Organ Failure Assessment.

### Microbial findings immediately after ECMO decannulation

Blood cultures obtained within 10 min after ECMO decannulation yielded positive results in 6 of 18 patients (33%; 95% CI: 13–59%); pathogen identification occurred after standard incubation (24–72 h) (Tables 4 and 5). In two of these cases (patients 12 and 13), the same pathogen was identified in blood cultures immediately before decannulation. Three patients (patients 2, 10, and 14) demonstrated previously undetected pathogens (Tables 4 and 5). In one patient (patient 17), the same pathogen that caused bacteremia during ECMO therapy reappeared (Tables 4 and 5). Among these, three patients harbored skin flora–associated pathogens, while the remaining three exhibited organisms of intestinal origin. Only one gram-positive bacterial strain and one fungal isolate were identified (Tables 4 and 5).

### Microbiological results of ECMO cannulas and swabs of insertion sites

In patient 15, *Staphylococcus* (*S.) epidermidis* was detected on the ECMO cannula (Tables 4 and 5). The patient had previously been diagnosed with *S. epidermidis* sepsis during ECMO support (Tables 4 and 5). At the time of ECMO decannulation, no antibiotic therapy was administered, and there were no clinical signs of bacteremia present. However, subsequent blood cultures were positive for *S. epidermidis*.

In patient 18, a smear test from the ECMO cannula insertion site revealed the presence of *Candida (C.) albicans* and *Enterococcus (E.) faecium* (Table 4). These pathogens were not identified in blood cultures obtained before or after ECMO, likely because of ongoing antifungal and antibiotic treatment. Follow-up microbiological tests yielded negative results.

**Table 4:** Conventional microbiological findings around ECMO decannulation.

Pt	Blood culture during ECMO	Blood culture after ECMO	Blood culture pre-decannulation	Blood culture post-decannulation	Cannula	Insertion site swab	Sepsis post-ECMO
1	*E. faecium*, *K. aerogenes*, *St. oralis*, *B. ovatus*, *B. xylanisolvens*	Negative	Negative	Negative	Negative	–	Yes
2	*S. epidermidis* (ORSE)	*S. epidermidis* (ORSE), E. faecium	Negative	E. faecium	–	–	No
3	*S. epidermidis* (ORSE), *E. faecium*	Negative	–	–	–	–	No
4	*S. haemolyticus*	*G. adiacens*, *A. viscosus*	Negative	Negative	–	–	No
5	Negative	*S. epidermidis* (ORSE)	Negative	Negative	Negative	–	Yes
6	*P. rettgeri*, *C. freundii* (4MRGN)	Negative	Negative	Negative	Negative	Negative	Yes
7	Negative	*S. epidermidis*, *S. warneri*, *S. hominis*, *E. coli*	Negative	Negative	–	–	Yes
8	*St. mitis*, *M. luteus*, *St. constellatus*, *N. flava*	Negative	Negative	Negative	Negative	–	No
9	*C. acnes*	*S. epidermidis*	Negative	Negative	Negative	–	No
10	Negative	*S. epidermidis* (ORSE), *S. capitis*	Negative	*S. capitis*	Negative	–	Yes
11	*Str. anginosus*, *St. mitis*	*K. pneumoniae*, *C. acnes*	Negative	Negative	Negative	–	Yes
12	Negative	*S. aureus* (MSSA)	*S. aureus* (MSSA)	*S. aureus* (MSSA)	–	–	Yes
13	Negative	*C. glabrata*	*C. glabrata*	*C. glabrata*	–	–	Yes
14	Negative	*Pr. mirabilis*	Negative	*Pr. mirabilis*	–	–	No
15	*S. epidermidis* (ORSE)	*S. epidermidis* (ORSE)	Negative	Negative	*S. epidermidis*	–	Yes
16	*A. junii*, *C. glabrata*	Negative	Negative	Negative	–	–	No
17	*S. caprae*	*S. epidermidis* (ORSE), *S. caprae*, *E. faecium* (VRE)	Negative	*S. caprae*	–	–	No
18	*C. albicans*, *C. glabrata*, *B. thetaiotaomicron*	Negative	Negative	Negative	Negative	*E. faecium*, *C. albicans*	Yes

Pt, patient number. 4MRGN, multidrug-resistant gram-negative with resistance to 4 antibiotic classes; A., Acinetobacter; B., Bacteroides; C., Candida/Cutibacterium; E., Enterococcus/Escherichia; G., Granulicatella; K., Klebsiella; M., Micrococcus; MSSA, methicillin-susceptible S. aureus; N., Neisseria; ORSE, oxacillin-resistant S. epidermidis; P., Providencia; Pr., Proteus; S., Staphylococcus; St./Str., Streptococcus; VRE, vancomycin-resistant Enterococcus. NGS detected additional organisms (patients 7, 10, 15, and 18) and missed culture-confirmed pathogens (patient 13, C. glabrata).

### Next generating sequencing results of plasma samples before and after decannulation

As described in the Methods section, to enrich for mcfDNA, human-derived sequences were removed by mapping reads against the GRCh38 human reference genome using NextGenMap, followed by extraction of unmapped reads. These non-human reads were then used for downstream taxonomic classification using Kraken2 with a comprehensive, custom-built database. Only species-level classifications with confidence scores ≥ 0.6 were retained. Post-classification analysis involved in-house Python and R scripts to compute the SIQ score, which reflects the pathogenic potential of the detected species relative to a control cohort of non-septic postoperative patients. This approach, closely following the original NextGeneSiS framework, enabled sensitive detection of bloodstream infections and supported clinically relevant interpretations of microbial signatures in patients with sepsis. In 9 of 12 patients (75%) with available samples (patients 7, 10, 12, 13, 14, 15, 16, 17, and 18; Tables 4 and 5), NGS detected bacterial, fungal, or viral DNA (DNAemia). In two of these cases (17%; patients 12 and 14), the findings were consistent with the conventional microbiological results.

In four patients (33%; patients 7, 16, 17, and 18), NGS identified bacterial and fungal pathogens that were also detected by conventional microbiological testing during ECMO. In five patients (42%; patients 7, 10, 15, 16, and 18), sequencing detected additional pathogens not identified by blood cultures, providing a possible explanation for post-decannulation sepsis in four cases (patients 7, 10, 15, and 18). In one patient (patient 13), NGS detected only viral material, while the fungus *Candida glabrata* was identified by blood culture. Interestingly, three patients (25%; patients 10, 15, and 18) exhibited NGS-detected bacterial pathogens without correlation to any microbiological findings during or after ECMO therapy in our ICU. In these cases, even PCR testing of blood (patients 10, 13, and 15) and bronchoalveolar lavage fluid (patients 10, 13, 15,18) upon ICU admission was negative (Table S5 in the Supplement).

Sequencing data for Patient 18 showed *Streptococcus pyogenes*, which had previously been isolated from blood cultures in an external hospital prior to ECMO initiation. Additionally, in the same patient, sequencing detected more *Bacteroides* species than conventional cultures. In patient 15, *Escherichia coli* was detected by sequencing; this pathogen was later found in tracheal fluid 21 days after ICU discharge at a rehabilitation facility. Notably, this patient had persistently elevated C-reactive protein levels. Patient 10 showed pathogens typical of ICU-acquired infections (*Staphylococcus aureus* and *Klebsiella pneumoniae*), although these organisms were not identified by routine microbiology. Furthermore, NGS identified viral pathogens in three patients (25%), of which only one case had been detected by standard virological diagnostics during the ICU stay. In the four patients who developed sepsis following ECMO decannulation (patients 7, 10, 15, and 18), metagenomics revealed pathogens that were not detected by conventional microbiology (Table 5).

**Table 5:** NGS results (mcfDNA) around ECMO decannulation.

Pt	NGS pre-decannulation	NGS post-decannulation	Virology during ICU stay	Concordance with culture
1	No data	No data	CMV reactivation	–
2	No data	No data	SARS-CoV-2	–
3	No data	No data	SARS-CoV-2, HSV-1, CMV	–
4	No data	No data	Negative	–
5	No data	No data	HSV-1 reactivation	–
6	No data	No data	SARS-CoV-2, HSV-1	–
7	*H. influenzae*	*H. influenzae*	SARS-CoV-2	Partial (*H. influenzae* in BAL)
8	Negative	Negative	HSV-1 reactivation	Concordant
9	Negative	Negative	CMV reactivation	Concordant
10	HHV-6B, *K. pneumoniae*, *S. aureus*	Negative	CMV reactivation	Discordant (not in culture)
11	Negative	Negative	HSV-1, CMV reactivation	Concordant
12	*S. aureus*	*S. aureus*	Negative	Concordant
13	EBV	EBV	Previous EBV	Discordant (*C. glabrata* missed)
14	Negative	*Pr. mirabilis*	Hep C	Concordant
15	Negative	*E. coli*	SARS-CoV-2	Discordant (*E. coli* not in culture)
16	*St. pyogenes*	*St. pyogenes*, *S. aureus*	CMV reactivation	Partial (*St. pyogenes* in BAL)
17	*S. caprae*	Negative	HSV-1	Inconclusive†
18	*B. ovatus*, *B. thetaiotaomicron*, *C. albicans*, *E. faecium*, CMV, EBV, *St. pyogenes*	*C. glabrata*, *B. ovatus*, *B. thetaiotaomicron*, *E. faecium*, CMV, EBV, *S. aureus*, *St. pyogenes*	CMV, EBV	Partial

NGS results are shown as organisms detected by mcfDNA sequencing. Pt, patient number. DNAemia indicates the detection of microbial cell-free DNA but does not confirm viable organisms. †Patient 17 concordance inconclusive. B., Bacteroides; C., Candida; CMV, cytomegalovirus; E., Escherichia/Enterococcus; EBV, Epstein-Barr virus; H., Haemophilus; HHV-6B, human herpesvirus 6B; HSV-1, herpes simplex virus 1; K., Klebsiella; Pr., Proteus; S., Staphylococcus; St., Streptococcus. NGS analysis was performed retrospectively and did not influence antimicrobial therapy during the study.

## Discussion

In our cohort, we observed a notably high incidence of sepsis (56%) following ECMO decannulation, with the majority of cases occurring within the first seven days. In our cohort, blood cultures post-decannulation yielded the same pathogen as previously identified during ECMO support in one patient, while NGS confirmed identical pathogens in three additional patients. A striking finding of our study was the high prevalence of bacteremia immediately following ECMO decannulation (33%) as detected by conventional microbiological testing and 75% among the subset of 12 patients analyzed using NGS.

In our study, *Staphylococcus epidermidis* was identified in the ECMO cannula but not in the corresponding blood cultures. Additionally, *Escherichia coli* was detected via sequencing in the plasma 21 days before it was isolated microbiologically in patient 15. These findings highlight the diagnostic limitations of culture-based methods in ECMO patients and suggest that biofilm-associated infections may evade detection unless ECMO cannulas are systematically analyzed post-removal^5,7,8,10^. Plasma cell-free DNA next-generation sequencing offers a promising solution by enabling the simultaneous detection of bacterial, fungal, and viral pathogens, as well as parasites^40,41^. This method circumvents many pitfalls of traditional diagnostics and significantly improves the detection rates in patients with sepsis by capturing microbial cfDNA fragments^42,43^. However, the enhanced sensitivity of NGS introduces challenges in interpretation. Detection of microbial DNA, particularly at low abundance, does not necessarily indicate a causative infection warranting antimicrobial therapy. Some NGS-positive findings may represent contamination, colonization, transient bacteremia, or residual DNA from previously treated infections, rather than active disease. For patients 7 and 15, the clinical plausibility of the detected pathogens was supported by concordance with prior microbiological findings (H. influenzae in BAL for patient 7) and subsequent clinical course (E. coli isolation 21 days later with persistently elevated CRP for patient 15). Nevertheless, we acknowledge that causation cannot be definitively established from these data. Therefore, rather than replacing conventional diagnostics, NGS should be viewed as a complementary tool that requires careful clinical interpretation of the results.

The detection of human herpesviruses in this study cohort was not unexpected, as reactivation of latent herpesviridae is a well-documented phenomenon in critically ill patients and has been associated with increased rates of intensive care admission, particularly in those with pulmonary infections^44^. Notably, human herpesviruses may also play a modulatory role in polymicrobial interactions, influencing the formation and stability of bacterial and fungal biofilms, especially those formed by *C. albicans* and *S. aureus*^45^. Moreover, *C. albicans* biofilms have been shown to provide a protective niche for herpesviruses, shielding them from antiviral therapies and potentially serving as a reservoir for persistent viral presence^46^. These interactions may have important implications for pathogen persistence and treatment resistance in patients undergoing ECMO and warrant further investigation. Human betaherpesviruses have been implicated in oral health, especially in the context of periodontal and peri-implant diseases, where they have been detected in subgingival biofilms^47,48^. These findings introduce important new perspectives for critically ill patients undergoing ECMO.

Conventional biomarkers, such as PCT and CRP, although widely used in clinical settings, demonstrate limited specificity and sensitivity in this context^49,50^. PCT is a well-established marker of bacterial infections and sepsis; however, its diagnostic accuracy varies^51,52^. In this small patient population, PCT levels did not differ significantly between patients with and without sepsis post-decannulation. Interestingly, PCT levels declined in both groups on the day following decannulation, which further complicates the interpretation. This finding may reflect the non-specific immune modulation induced by ECMO therapy^11,53^. Some evidence suggests that combining PCT with other biomarkers, such as albumin levels and the neutrophil-to-lymphocyte ratio, can enhance diagnostic accuracy in critically ill patients^54^. Therefore, while PCT remains a useful biomarker, it should not be used in isolation but rather integrated into a broader diagnostic framework^55,56^.

Our findings highlight that integrating advanced molecular diagnostic techniques, such as NGS, with conventional clinical and microbiological assessments may significantly enhance the early detection and characterization of sepsis in patients undergoing ECMO. Moreover, the development of rapid, point-of-care testing platforms with integrated antimicrobial resistance testing could enable more timely therapeutic interventions, ultimately improving patient outcomes and reducing mortality^57,58^.

### Limitations

This study has several limitations. The small sample size reflects the challenges inherent in prospective ECMO research, including high mortality (54%) and consent requirements. These findings require multicenter validation. The 95% confidence interval for the observed 56% sepsis rate (31–79%) underscores this uncertainty. The single-center design limits the generalizability of the results. NGS was performed retrospectively on stored plasma and did not influence the clinical decisions. NGS-culture discordance occurred in both directions: NGS detected additional organisms missed by culture but also failed to detect *C. glabrata* identified by culture in one patient. Blood sampling was not standardized relative to antibiotic dosing, and 94% of patients received antibiotics at decannulation, which may have affected the culture yield. Our findings are associative and cannot establish a causal relationship between biofilm dislodgement and post-decannulation sepsis. The interpretation of NGS results requires careful clinical judgment. The increased sensitivity of molecular methods may detect microbial DNA that does not represent active infection, including contamination, colonization, or residual DNA from treated infections. Not all NGS-detected organisms warrant antimicrobial therapy, and clinical correlation is essential for appropriate management decisions. The interaction between anticoagulation strategies and biofilm formation in ECMO circuits warrants further investigation. While anticoagulation is essential to maintain circuit patency and prevent thrombotic complications, its potential influence on microbial colonization and biofilm development remains incompletely understood.

Despite these limitations, this study had notable strengths. This is the first prospective study to specifically examine bacteremia and sepsis at ECMO decannulation. The prospective design with protocolized sampling at defined timepoints (pre-decannulation and within 10 min post-decannulation), systematic cannula tip analysis, and integration of conventional and molecular diagnostics provide methodological rigor not achievable in retrospective analyses. The within-subject comparison (pre- vs. post-decannulation) minimizes inter-individual confounding. The standardized 7-day follow-up systematically captured the critical post-decannulation period. These data provide a foundation for larger multicenter validation study.

## Conclusions

Our study revealed a high incidence of septic complications after ECMO decannulation, with challenges in immediate recognition using gold-standard microbiology. The presence of pathogens on ECMO cannulas and insertion sites suggests that biofilm formation and catheter-related infections may contribute to sepsis development. These findings highlight the need for close monitoring and management of patients after ECMO decannulation to prevent and treat septic complications. However, subsequent validation studies in larger cohorts are necessary to fully ascertain whether biofilm-induced BSI is responsible for sepsis after ECMO initiation. Our study provides a foundation for future investigations into the mechanisms underlying the increased susceptibility to infections in patients on ECMO and the development of targeted interventions to improve outcomes in this critically ill population.

## Supplementary Information

Below is the link to the electronic supplementary material.

Supplementary Material 1

## Data Availability

The datasets generated and/or analysed during the current study are available in the European Nucleotide Archive (ENA) repository, accession number PRJEB100700, [https://www.ebi.ac.uk/ena/browser/view/PRJEB100700](https:/www.ebi.ac.uk/ena/browser/view/PRJEB100700) .

## Abbreviations

4MRGN: Multidrug-resistant gram-negative bacteria (resistance to 4 antibiotic classes)
aPTT: Activated partial thromboplastin time
ARDS: Acute respiratory distress syndrome
BAL: Bronchoalveolar lavage
BMI: Body mass index
BSI: Bloodstream infection
C.: Candida
cfDNA: Cell-free DNA
CI: Confidence interval
CMV: Cytomegalovirus
COVID-19: Coronavirus disease 2019
CRP: C-reactive protein
DNA: Deoxyribonucleic acid
E.: *Escherichia*
EBV: Epstein-Barr virus
ECLS: Extracorporeal life support
ECMO: Extracorporeal membrane oxygenation
ELSO: Extracorporeal Life Support Organization
En.: *Enterococcus*
HHV-6B: Human herpesvirus 6B
HSV-1: Herpes simplex virus 1
ICU: Intensive care unit
IQR: Interquartile range
K.: Klebsiella
mcfDNA: Microbial cell-free DNA
MSSA: Methicillin-susceptible *Staphylococcus aureus*
NGS: Next-generation sequencing
ORSE: Oxacillin-resistant *Staphylococcus epidermidis*
P.: *Pseudomonas*
PCR: Polymerase chain reaction
PCT: Procalcitonin
Pr.: Proteus
QC: Quality control
RASS: Richmond agitation-sedation scale
S.: *Staphylococcus*
SARS-CoV-2: Severe acute respiratory syndrome coronavirus 2
SEM: Standard error of the mean
SIQ: Sepsis indicating quantifier
SOFA: Sequential organ failure assessment
St./Str.: *Streptococcus*
VA-ECMO: Veno-arterial extracorporeal membrane oxygenation
VRE: Vancomycin-resistant *Enterococcus*
VV-ECMO: Veno-venous extracorporeal membrane oxygenation
