# Supplementary material for: Prevalence and microbiological profile of septic complications following ECMO decannulation: a prospective single-center study
Source: Sci Rep. 2025 Dec 29;15:45668. doi: 10.1038/s41598-025-34085-1 (PMC12753795; doi:10.1038/s41598-025-34085-1)
Supplement: Supplementary file 1 — Supplementary Material 1 [file 41598_2025_34085_MOESM1_ESM.docx]

**SUPPLEMENTAL MATERIAL**

**TABLE S1.** Inclusion and exclusion criteria of the present study.

| **Inclusion criteria** | **Exclusion criteria** |
| --- | --- |
| - Written informed consent - Age ≥ 18 years - Venovenous or venoarterial ECMO therapy | - Refusal to participate in the study - Age < 18 years |

**TABLE S2.** Recorded data.

| **Patients Characteristics** | **Laboratory parameters for eight days** |
| --- | --- |
| **Age**, yrs. | **Leucocyte**, ×10^3^/µl |
| **Gender**, M/F | **Thrombocytes**, /nl |
| **BMI** kg/m^2^ | **Total Serum Bilirubin**, mg/dl |
| **Pre-existing conditions:** | **Creatinine,** mg/dl |
| **diseases** | **CRP**, mg/dl |
| **lung diseases** | **PCT**, ng/ml |
| **Immunosuppression medication** | **Lactate,** mmol/l |
| **Duration of** |  |
| **intubation pre ECMO** (days) | **Clinical Parameters for eight days** |
| **ECMO therapy** (days) | **Norepinephrine infusion,** µg/kgKG/min |
| **ICU stay** (days) | **p_a_O_2_/F_i_O_2_ratio** |
| **Reasons for acute respiratory failure** | **Body temperature,** °C |
| **ARDS** | **SOFA Score** |
| **Primary viral pneumonia** | **Sedation (RASS)** |
| **Primary bacterial pneumonia** |  |
| **Status Asthmaticus** |  |
| **Other reason for ECMO** |  |
| **Infections** |  |
| **Sepsis pre ECMO therapy** |  |
| **Sepsis during ECMO therapy** |  |
| **Sepsis pneumogenic** |  |
| **Antibiotics pre ECMO therapy** |  |
| **Antibiotics during ECMO therapy** |  |
| **Antibiotics after ECMO therapy for eight days** |  |
| **Microbiological evidence** |  |
| **Bacterial detection pre ECMO therapy** |  |
| **Bacterial detection during ECMO therapy** |  |
| **Bacterial detection after ECMO therapy** |  |
| **Fungal detection pre ECMO therapy** |  |
| **Fungal detection during ECMO therapy** |  |
| **Fungal detection after ECMO therapy** |  |
|  | |

**TABLE S3.** Conversion of Richmond Agitation and Sedation Scale into Glasgow Coma Scale for SOFA Score by Vasilevskis et al. 2016 (DOI: 10.1097/CCM.0000000000001375).

| **Richmond Agitation and Sedation Scale** | **Glasgow Coma Scale** | **Neurological System in SOFA Score** |
| --- | --- | --- |
| ≥ 0 | 15 | 0 |
| -1 | 13-14 | 1 |
| -2 | 10-12 | 2 |
| -3 | 6-9 | 3 |
| ≤ -4 | 3-5 | 4 |
| Legend: SOFA: Sequential Organ Failure Assessment. | | |

**TABLE S4.** Assessment for SOFA Score.

| **Parameter** | **Day x** | **Day x+1** | **p-value** |
| --- | --- | --- | --- |
| Respiratory System (p_a_O_2_/F_i_O_2_ ratio) | 4 | 2 [1-3] | .001 |
| Neurological System (sedation) | 3 [2 – 4] | 1 [0 - 3] | .05 |
| Cardiovascular System (norepinephrine dose) | 0 | 3 [0-3] | .06 |
| Coagulation (platelets) | 2 [1-2] | 1 [0-2] | .03 |
| Liver (bilirubin) | 0 [0-1] | 0 [0-1] | 1 |
| Renal Function (creatinine) | 1 [0-4] | 1 [0-4] | 1 |
| Legend: not normally distributed data presented as median [25.quartile - 75. quartile]. p-values obtained by Wilcoxon test. | | | |

**TABLE S5.** Multiplex PCR on ICU admission.

| **Substance** | **ethylenediaminetetraacetic acid blood** | **Bronchioalveolar fluid** |
| --- | --- | --- |
| **Pathogens** | Staphylococcus aureus  Streptococcus pneumoniae  Enterococcus faecium  Enterococcus faecalis  Escherichia coli  Klebsiella pneumoniae  Klebsiella oxytoca  Klebsiella aerogenes  Enterobacter cloacae complex  Serratia marcescens  Pseudomonas aeruginosa  Stenotrophomonas maltophilia  Candida albicans  Candida glabrata | Staphylococcus aureus  Streptococcus pneumoniae  Escherichia coli  Enterobacter cloacae complex  Klebsiella aerogenes  Proteus sp.  Morganella morganii  Serratia marcescens  Citrobacter freundii  Klebsiella pneumoniae  Klebsiella oxytoca  Klebsiella variicota  Pseudomonas aeruginosa  Acinetobacter baumannii complex  Legionella pneumophila  Moraxella catarrhalis  Stenotrophomonas maltophilia  Haemophilus influenzae  Chlamydophila pneumoniae  Pneumocystis jiroveci  Mycoplasma pneumoniae |
